# The miR-35-41 Family of MicroRNAs Regulates RNAi Sensitivity in *Caenorhabditis elegans*

**DOI:** 10.1371/journal.pgen.1002536

**Published:** 2012-03-08

**Authors:** Katlin B. Massirer, Saida G. Perez, Vanessa Mondol, Amy E. Pasquinelli

**Affiliations:** Division of Biology, University of California San Diego, La Jolla, California, United States of America; University of California San Francisco, United States of America

## Abstract

RNA interference (RNAi) utilizes small interfering RNAs (siRNAs) to direct silencing of specific genes through transcriptional and post-transcriptional mechanisms. The siRNA guides can originate from exogenous (exo–RNAi) or natural endogenous (endo–RNAi) sources of double-stranded RNA (dsRNA). In *Caenorhabditis elegans*, inactivation of genes that function in the endo–RNAi pathway can result in enhanced silencing of genes targeted by siRNAs from exogenous sources, indicating cross-regulation between the pathways. Here we show that members of another small RNA pathway, the mir-35-41 cluster of microRNAs (miRNAs) can regulate RNAi. In worms lacking miR-35-41, there is reduced expression of *lin-35/Rb*, the *C. elegans* homolog of the tumor suppressor Retinoblastoma gene, previously shown to regulate RNAi responsiveness. Genome-wide microarray analyses show that targets of endo–siRNAs are up-regulated in *mir-35-41* mutants, a phenotype also displayed by *lin-35/Rb* mutants. Furthermore, overexpression of *lin-35/Rb* specifically rescues the RNAi hypersensitivity of *mir-35-41* mutants. Although the *mir-35-41* miRNAs appear to be exclusively expressed in germline and embryos, their effect on RNAi sensitivity is transmitted to multiple tissues and stages of development. Additionally, we demonstrate that maternal contribution of miR-35-41 or *lin-35/Rb* is sufficient to reduce RNAi effectiveness in progeny worms. Our results reveal that miRNAs can broadly regulate other small RNA pathways and, thus, have far reaching effects on gene expression beyond directly targeting specific mRNAs.

## Author Summary

RNA interference (RNAi) has become a widely used approach for silencing genes of interest. This tool is possible because endogenous RNA silencing pathways exist broadly across organisms, including humans, worms, and plants. The general RNAi pathway utilizes small ∼21-nucleotide RNAs to target specific protein-coding genes through base-pairing interactions. Since RNAs from exogenous sources require some of the same factors as endogenous small RNAs to silence gene expression, there can be competition between the pathways. Thus, perturbations in the endogenous RNAi pathway can result in enhanced silencing efficiency by exogenous small RNAs. MicroRNAs (miRNAs) comprise another endogenous small RNA pathway, but their biogenesis and mechanism of gene silencing are distinct in many ways from RNAi pathways. Here we show that a family of miRNAs regulates the effectiveness of RNAi in *Caenorhabditis elegans*. Loss of *mir-35-41* results in enhanced RNAi by exogenous RNAs and reduced silencing of endogenous RNAi targets. The embryonic miR-35-41 miRNAs regulate the sensitivity to RNAi through *lin-35/Rb*, a homolog of the human Retinoblastoma tumor suppressor gene previously shown to regulate RNAi effectiveness in *C. elegans*. Additionally, we show that this sensitivity can be passed on to the next generation of worms, demonstrating a far-reaching effect of the miR-35-41 miRNAs on gene regulation by other small RNA pathways.

## Introduction

The ability of double stranded RNA (dsRNA) to induce silencing of specific genes was discovered in the nematode *Caenorhabditis elegans* and dubbed RNA interference (RNAi) [1]. RNAi was subsequently identified in many organisms, including mammals, providing a powerful experimental tool for inactivating specific genes [2], [3]. In worms, RNAi can be initiated by long dsRNAs from exogenous sources, such as injected or ingested bacterially produced dsRNA [1], [4]. Cellular factors process the dsRNAs into small interfering RNAs (exo–siRNAs) of ∼21 nucleotides long, which target complementary mRNAs for degradation [5]. Exo–siRNAs form complexes with Argonaute proteins that mediate target mRNA cleavage or degradation through other mechanisms [6]. Recently, a nuclear RNAi pathway was discovered in worms where the Argonaute NRDE-3 silences target genes through a co-transcriptional mechanism of silencing [7], [8].

Comparable to exo–RNAi, endogenous dsRNAs can enter processing pathways that produce endo–siRNAs that silence target genes through base-pairing interactions [6], [9]. Endo–siRNAs have been identified by small RNA cloning in *C. elegans*, Drosophila and mouse and are predicted to target thousands of endogenous genes, particularly mRNAs present in germline and embryos [10], [11], [12], [13], [14], [15]. In *C. elegans*, both the exo- and endo–siRNA pathways require the endoribonuclease enzyme Dicer (DCR-1) to process the initiating dsRNAs into ∼21 nt siRNAs, but distinct Argonaute proteins usually bind the different types of siRNAs to mediate gene silencing [16], [17]. For example the Argonaute RDE-1 is required for RNAi initiated by exogenous but not endogenous dsRNAs [17].

In nematodes and plants, RNAi-mediated silencing can be amplified through the production of secondary siRNAs by RNA dependent RNA polymerases (RdRPs). In *C. elegans*, loss of the RdRP RRF-3 results in greatly reduced levels of endo–siRNAs and an enhanced response to exogenous dsRNA 18,19. Likewise, the exonuclease ERI-1 is required for the accumulation of some endo–siRNAs and worms with mutations in *eri-1* display hypersensitivity to exo–siRNAs [18], [20]. Another class of mutants with enhanced RNAi is represented by *lin-35/Rb*, which encodes the worm homolog of the Retinoblastoma tumor suppressor, and includes other genes in the *lin-35/Rb* pathway such as *lin-15*, *dpl-1* (mammalian DP), *and hpl-2* (mammalian HP1) [21], [22], [23]. The molecular mechanism by which *lin-35/Rb* negatively regulates the RNAi pathway remains to be fully understood. Worms with mutations in *lin-35/Rb* have up-regulated levels of mRNAs corresponding to cloned endo–siRNAs, suggesting decreased levels or function of endo–siRNAs in these mutants [24]. Reduced endo–RNAi activity may free limiting factors for the exo–RNAi pathway. Furthermore, some of the up-regulated genes in *lin-35/Rb* mutants encode Argonaute proteins that might also contribute to enhanced exo–RNAi [24]. Additionally, *lin-35/Rb* mutants have increased expression of germline-specific genes in somatic tissues [23]. This expression pattern is predicted to enhance RNAi sensitivity by providing factors normally utilized in the germline for silencing pathways [23].

MicroRNAs (miRNAs) represent another class of small RNAs that derive from endogenously expressed transcripts [25]. Typically, miRNAs are transcribed as long primary transcripts containing hairpin structures that are released by Drosha cleavage. The resulting precursor undergoes processing by Dicer to produce the mature ∼22 nt miRNA, which forms a complex with Argonaute proteins. While mammalian miRNAs seem to distribute evenly among the four Argonaute proteins, worm and fly miRNAs mostly associate with specific Argonautes [6]. For example, Argonaute Like Genes 1 and 2 (ALG-1/2) bind worm miRNAs, but not exo- or endo–siRNAs [26]. In contrast to exo and endo–siRNAs, animal miRNAs pair with imperfect sequence complementarity to their target mRNAs, causing target degradation and translational repression [27]. The common factor for most small RNA pathways is Dicer. Loss of Dicer activity in worm or mammalian cells results in defective RNAi and severely reduced levels of most miRNAs and endo–siRNAs [16], [18], [28], [29], [30]. However, mutations in other genes required for endo- or exo–RNAi seem to have little effect on the miRNA pathway in *C. elegans* [16], [18].

In an effort to understand the function of specific miRNAs, a large collection of deletion mutants was generated in *C. elegans* [31]. While most individual mutants displayed no obvious phenotypes, simultaneous deletion of multiple family members (miRNAs with identical sequences at positions 2–7) often resulted in developmental defects and lethality [32], [33]. For example, the miR-35-41 miRNAs are clustered within ∼800 nt of a common transcript and share a high degree of sequence homology [34]. Deletion of this miRNA cluster results in defective germ and intestinal cell proliferation and temperature sensitive embryonic lethality [32], [35]. While the genes regulated by miR-35-41 that are responsible for these phenotypes are yet to be determined, several direct targets of these miRNAs were recently validated in embryonic extracts [36]. Although the miR-35-41 miRNAs have only been found in worms and planaria [34], [37], poorly conserved miRNA clusters have also been observed to be highly expressed and function in early development in other species, including Drosophila, mouse and humans [38], [39], [40].

Here we show that the miR-35-41 miRNAs regulate other small RNA pathways in *C. elegans*. Deletion of the miRNA cluster results in worms with enhanced RNAi sensitivity in multiple developmental stages and tissues. This effect is likely related to decreased endo–RNAi activity in *mir-35-41* mutants, as they also exhibit up-regulation of many endo–siRNA targets. We found that the miR-35-41 miRNAs negatively regulate the exo–RNAi pathway through *lin-35/Rb* and that this function can be supplied maternally to the progeny. These results point to a new level of cross-regulation between small RNA pathways, whereby the expression of specific miRNAs can impact the efficiency of RNAi mediated by exo– and endo–siRNAs.

## Results

### Enhanced RNAi in *mir-35-41* mutants

**Figure 1:**
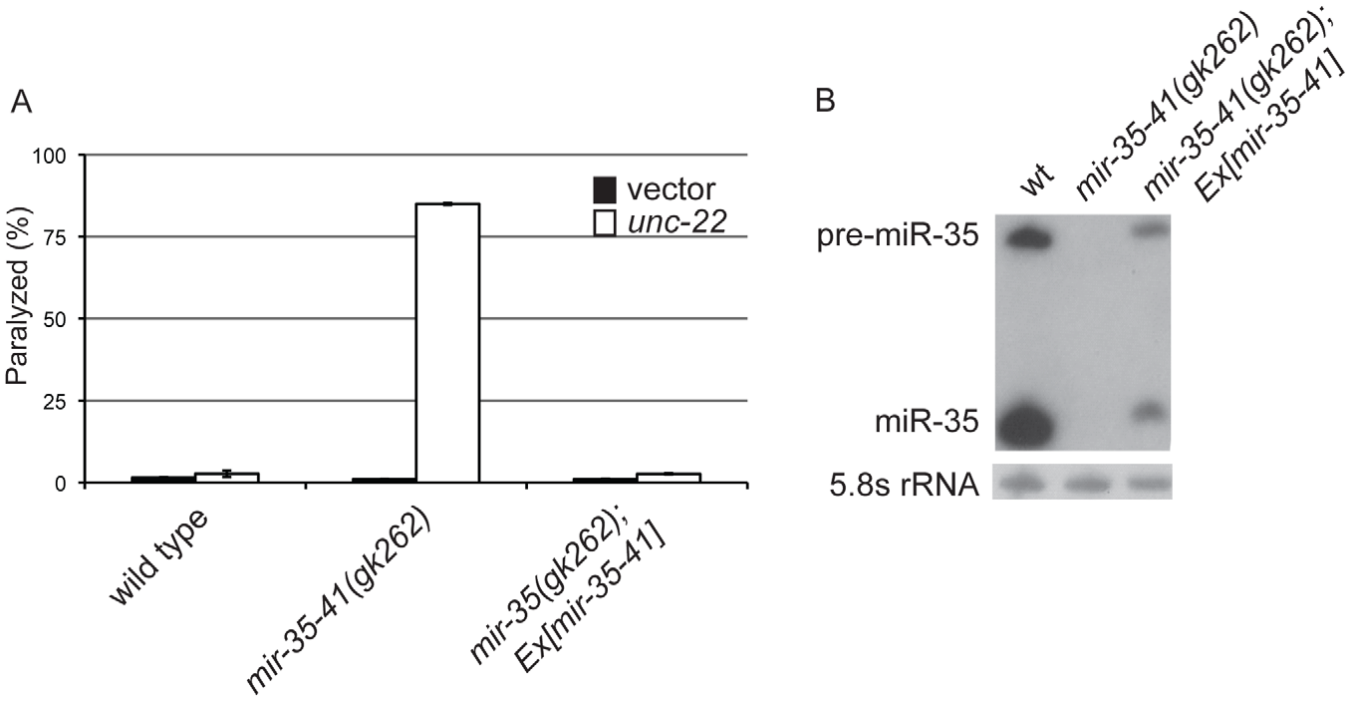
Deletion of the *mir-35-41* miRNA cluster results in RNAi hypersensitivity. (A) L4 stage wild type, *mir-35-41(gk262)* and *mir-35-41(gk262)*; *apEx160 [mir-35-41]* strains were grown on bacteria expressing *unc-22* dsRNA (open columns) or the empty RNAi vector L4440 (black columns). The percentages of paralyzed worms after 28 h of exposure to RNAi conditions are graphed as the mean and standard deviation from three independent experiments. (B) PAGE Northern blot analysis of RNA from wild type, *mir-35-41(gk262)* and *mir-35-41(gk262)*; apEx160 [mir-35-41] embryos shows restored expression of precursor and mature *mir-35* miRNA in the transgenic strain. 5.8S rRNA levels are shown as loading controls.

In *C. elegans* the *mir-35-41* miRNAs are required for embryonic viability and proliferation of certain cell types, but the targets relevant for these phenotypes have not yet been established [31], [32], [35]. In the *mir-35-41(gk262)* and *mir-35-41(nDf50)* strains all seven members of the miRNA cluster are deleted, resulting in temperature sensitive embryonic lethality (Figure S1) [31], [32]. While using RNAi to identify factors that genetically interact with *mir-35-41(gk262)*, we discovered that this strain exhibits enhanced sensitivity to RNAi. In agreement with previous studies [1], [4], wild type worms fed bacteria expressing double stranded RNA targeting the muscle gene *unc-22* resulted in a high penetrance of worms with the twitching phenotype, while only 2% resembled the null *unc-22* loss-of-function phenotype of paralysis (Figure 1A and Table 1) [41]. In contrast, the majority (83%) of *mir-35-41(gk262)* worms were paralyzed by *unc-22*(RNAi) (Figure 1A and Table 1). This same treatment produced paralysis in about 20% of the established RNAi hypersensitive strain, *rrf-3(pk1426)* (Table 1), consistent with previous studies [19], [20]. We confirmed that the enhanced RNAi phenotype of *mir-35-41(gk262)* was due to loss of the miRNA gene instead of the overlapping anti-sense Y62F5A.9 protein-coding gene. A transgene encoding only the *mir-35-41* locus rescued the hypersensitivity of this strain to *unc-22*(RNAi) (Figure 1A) and supported miRNA expression in *mir-35-41(gk262)* worms (Figure 1B).

**Table 1:** The *mir-35-41(gk262)* mutant worms show enhanced RNAi in multiple tissues and stages of development.

Gene	RNAi phenotype	Worm strains			Tissue
		wt	*rrf-3(pk1426)*	*mir-35-41(gk262)*	
*unc-22*	% Prl*	2±2 (629)	17±5 (594)	83±14(651)	muscle
*lin-1*	% Muv†	1±0 (867)	54±7 (931)	69±8 (580)	hypodermal
*sqt-1*	% Rol†	7±1 (120)	80±4 (243)	84±6 (220)	hypodermal
*unc-86*	% Prl*	14±3 (173)	36±6 (354)	80±2 (291)	neuronal
*pos-1*	% Emb††	10±4 (248)	100±2 (456)	100±1 (513)	germline
*sex-1*	% Emb††	36±11 (298)	98±1 (522)	100±0 (832)	embryonic

Worm strains of the indicated genotype were grown on bacteria expressing dsRNA against the indicated genes at 20°C. RNAi phenotype results are mean ± standard deviation (average of 3 independent experiments). Numbers in parentheses represent the total number of adult worms or embryos scored.

*: L4 staged worms were transferred to RNAi and the percentage of paralyzed (%Prl) adult worms was scored 28 hours later.

**†:** L1 staged worms were plated on RNAi and the percentage of adult worms showing multivulva (%Muv) or roller (% Rol) phenotypes was scored.

**††:** L4 staged worms were transferred to RNAi, allowed to lay embryos and adult worms were removed 28 hours later. Percent embryonic lethal (%Emb) was calculated as the number of embryos that did not hatch.

Loss of *mir-35-41* results in enhanced RNAi in multiple tissues and stages of development. We observed that RNAi knockdown of *lin-1* (hypodermal), *sqt-1* (hypodermal), *unc-86* (neuronal), *pos-1* (germline) and *sex-1* (embryonic) resulted in enhanced phenotypes in *mir-35-41(gk262)* relative to wild type worms (Table 1). Furthermore, the effects were similar or stronger in *mir-35-41(gk262)* compared to the RNAi hypersensitive *rrf-3* mutants. These results indicate that expression of the miR-35-41 miRNAs negatively impacts the efficiency of exo–RNAi in *C. elegans*.

To determine if the enhanced RNAi phenotype of *mir-35-41(gk262)* was dependent on core exo–RNAi factors, we crossed this strain to mutants in the RNAi pathway. Upon *unc-22*(RNAi) treatment, the single mutants defective in RNAi (*rde-1*, *rde-4* and *rrf-1*) showed no phenotypic response, as expected (Table 2) [42], [43], [44]. Likewise, addition of mutations in *rde-1*, *rde-4* or *rrf-1* rendered *mir-35-41(gk262)* mutants completely RNAi defective (Table 2). These data show that *mir-35-41(gk262)* mutants require *rde-1*, *rde-4* and *rrf-1* to exhibit an RNAi response. While this might be expected, there is precedence for an RNAi hypersensitive strain (*lin-15B(n744)*) being independent of *rrf-1* activity [23].

**Table 2:** The RNAi sensitivity of *mir-35-41(gk262)* mutant worms is dependent on core RNAi factors.

Worm strains	Phenotype %		
	Unaffected	Twitching	Paralyzed
wild type (629)	10±1	88±3	2±2
*rde-1(ne300)* (282)	100	0	0
*rde-4(ne301)* (142)	100	0	0
*rrf-1(pk1417)* (254)	100	0	0
*mir-35-41(gk262)* (651)	0	17±5	83±14
*mir-35-41(gk262);rde-1(ne300)* (105)	100	0	0
*mir-35-41(gk262);rde-4(ne301)* (112)	100	0	0
*mir-35-41(gk262);rrf-1(pk1417)* (187)	100	0	0

L4 staged worms of each strain were transferred to *unc-22* RNAi and the percentage of unaffected, twitching or paralyzed adult worms was scored 28 hours later. Results are mean ± standard deviation (average of at least 2 experiments). Numbers in parentheses following the genotype represent the total number of worms scored.

**Figure 2:**
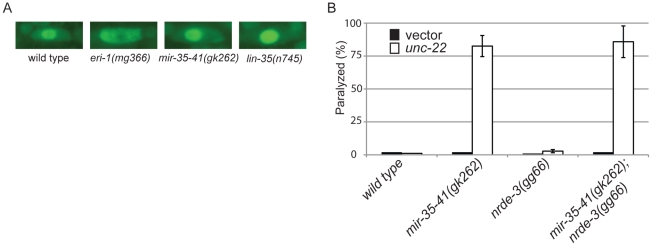
The RNAi hypersensitivity of *mir-35-41* mutants is independent of *nrde-3* activity. (A) Fluorescent microscopy showing subcellular localization of GFP::NRDE-3 in seam cells of the indicated genotypes. Pictures are representative of 50 worms analyzed for each strain. (B) Histogram representing the percentages of paralyzed worms for the indicated strains fed *unc-22* dsRNA for 28 hours from the L4 to adult stage. The means and standard deviations from three independent experiments are graphed.

The response of *mir-35-41(gk262)* to *unc-22*(RNAi) is comparable to that of the exceptionally RNAi hypersensitive strain *lin-35(n745)*, which carries a putative null mutation [21], [22], [23], [45]. About 20–30% of the population of the enhanced RNAi strains *rrf-3(pk1426)*, *eri-1(mg366)* and *ergo-1(tm1860)* exhibited paralysis in response to *unc-22*(RNAi) (Table 3). In comparison, over 80% of the *mir-35-41(gk262)* and *lin-35(n745)* strains were paralyzed by this RNAi treatment (Table 3). The *rrf-3*, *eri-1* and *ergo-1* genes are part of the endogenous RNAi pathway in *C. elegans* and loss of these genes results in decreased levels of endo–siRNAs [16], [17], [18], [46]. Diminished activity of the endo–RNAi pathway is one explanation for enhanced RNAi sensitivity to dsRNA from exogenous sources. The Argonaute protein NRDE-3 serves as a sensor for endo–siRNAs, as nuclear localization of this protein is dependent on the presence of siRNAs [8]. In contrast to wild type worms that express endo–siRNAs, GFP::NRDE-3 was mostly cytoplasmic in the seam cells of *eri-1(mg366)* worms, which are defective in endo–siRNA accumulation (Figure 2A) [8], [16], [18]. As in wild type, localization of GFP::NRDE-3 was nuclear in the *mir-35-41(gk262)* and *lin-35(n745)* mutant strains (Figure 2A). Additionally, the hypersensitivity of *mir-35-41* mutants continued in the absence of *nrde-3* activity (Figure 2B). These results show that in *mir-35-41* mutants sufficient endo–siRNAs are present to target NRDE-3 to the nucleus and the enhanced RNAi phenotype of these mutants does not require NRDE-3 activity.

**Table 3:** The RNAi hypersensitivity of *mir-35-41*(*gk26*2) is comparable to that of *lin-35(n745)*.

Worm strains	Phenotype %		
	Unaffected	Twitching	Paralyzed
wild-type (629)	10±1	88±3	2±2
*rrf-3(pk1426)* (594)	0	83±14	17±5
*eri-1(mg366)* (343)	0	73±3	27±3
*ergo-1(tm1860)* (452)	0	66±4	34±6
*lin-35(n745)* (248)	0	5±2	95±7
*mir-35(gk262)* (651)	0	17±5	83±14

L4 staged worms of each strain were transferred to *unc-22* RNAi and the percentage of unaffected, twitching or paralyzed adult worms was scored 28 hours later. Results are mean ± standard deviation (average of at least 2 experiments). Numbers in parentheses represent the total number of worms scored.

### Up-regulation of endogenous siRNA targets in *mir-35-41* mutants

**Figure 3:**
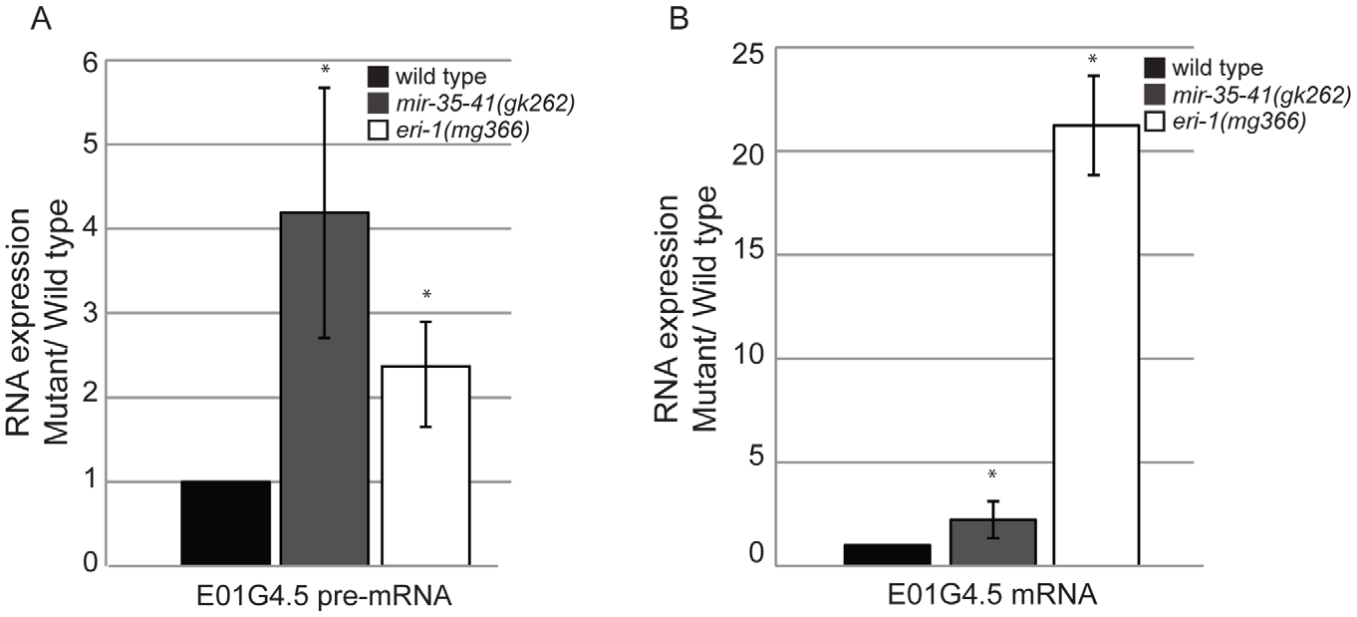
Mis-regulation of the E01G4.5 endo–siRNA target in *mir-35-41* mutants. RT-qPCR of E01G4.5 pre-mRNA (A) and mature mRNA (B) normalized to 18S rRNA and compared to wild type levels (mean ± s.e.m., n = 3, *, P<0.05).

To gain insight into the role of the *mir-35-41* miRNAs in regulating the exo–RNAi pathway, we performed microarray analysis of gene expression in WT versus *mir-35-41(gk262)* embryos. We chose statistically significant changes in gene expression of at least 1.5-fold differences between the triplicate sample averages (p value≤0.005 (two-sample t-test)) (Table S1). Of the 550 up-regulated genes in *mir-35-41(gk262)*, only a small fraction (4%) were predicted miR-35 targets based on a list of 606 genes from four different algorithms (Targetscan, PicTar, RNA22 and MiRWIP). Furthermore, obvious genes that might explain the heightened RNAi sensitivity of *mir-35-41(gk262)* were not mis-regulated at the mRNA level. Comparing the microarray results to a list of 6,469 endo–siRNA targets obtained from four independent studies revealed significant enrichment of endo–siRNA targets in the up-regulated genes (37%, p value 1.3e-05 two tailed exact Fisher's significance test) (Table S1) [11], [13], [18], [47]. In contrast, endo–siRNA targets were under-represented in the list of genes down-regulated in *mir-35-41(gk262)* (13% p value 2.2e-16 exact Fisher's significance test) (Table S1). Differential expression of the established endo–siRNA target E01G4.5 was confirmed by RT-qPCR (Figure 3). Similar to the previously reported effect of *eri-1* on this gene, both the spliced and unspliced forms of E01G4.5 were up-regulated in *mir-35-41* mutants (Figure 3) [8].

### Decreased LIN-35/Rb contributes to the RNAi hypersensitivity of *mir-35-41* mutants

**Figure 4:**
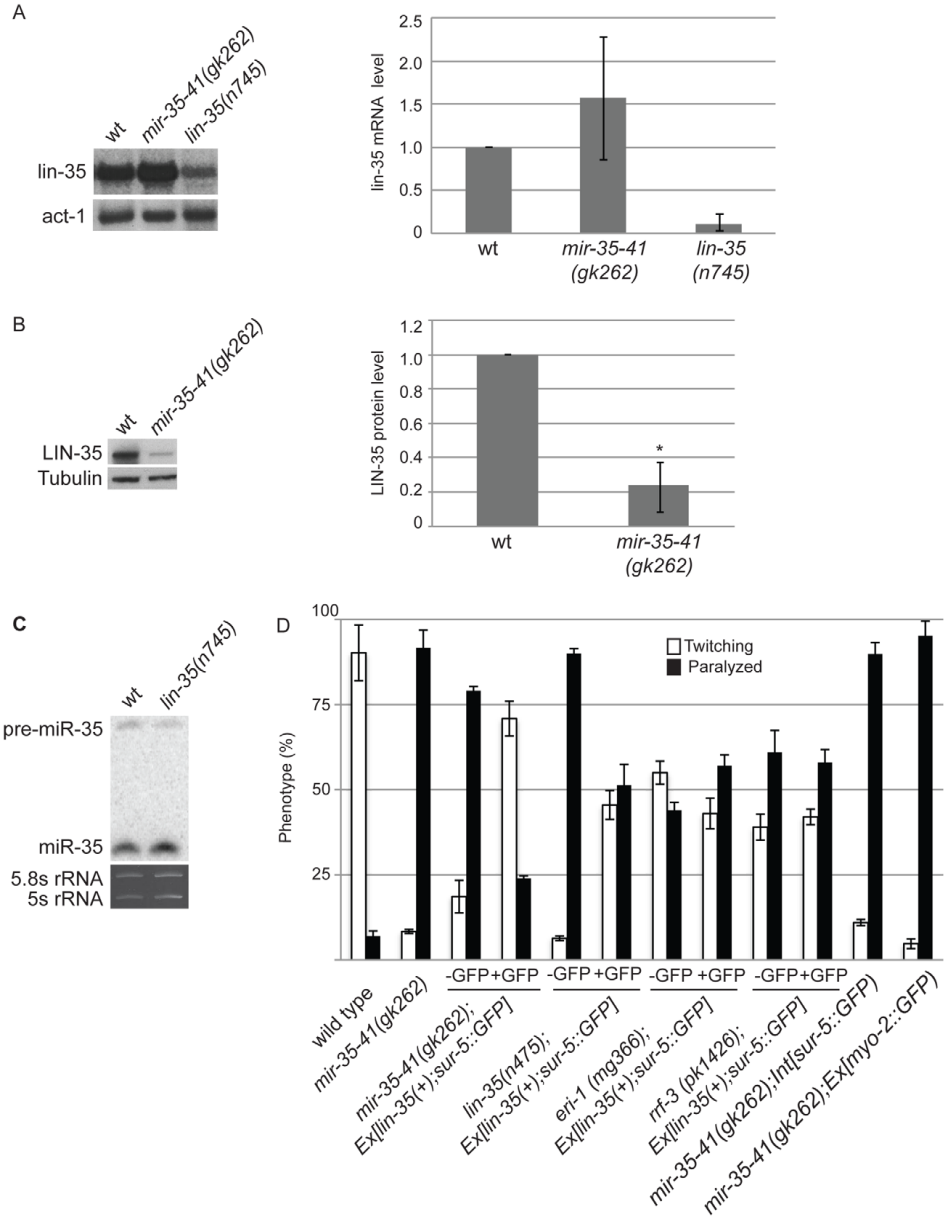
Decreased LIN-35/Rb contributes to the RNAi hypersensitivity of *mir-35-41(gk262)* worms. (A) Northern blot analyses of *lin-35/Rb* mRNA levels in WT, *mir-35-41(gk262)* and *lin-35(n745)* embryos. After normalization to actin mRNA the average and standard deviation from 3 independent experiments was calculated with wild type levels set to one. (B) LIN-35 protein is decreased in *mir-35-41(gk262)* embryos compared to wild type, as shown by western blotting. After normalization to tubulin the average and standard deviation from 4 independent experiments was calculated with wild type levels set to one (Student's t-test *p<4×10^−5^). (C) PAGE Northern blot analysis of RNA from wild type and *lin-35(n475)* embryos shows similar levels of pre- and mature miR-35 expression. The rRNAs are shown as loading controls. Results are representative of 3 independent experiments. (D) The indicated worm strains were grown on bacteria expressing *unc-22* dsRNA. *Ex[lin-35; sur-5::GFP]* is an extrachromosomal array that expresses *lin-35* in GFP positive (+) worms. *Int[sur-5::GFP]* is an integrated array that expresses GFP in all worms and *Ex[myo-2::GFP]* is an extrachromosomal array that expresses GFP in worms that inherit the array. Phenotype was scored as percent of twitching or paralyzed worms after 28 h of exposure to RNAi from the L4 to adult stage. Error bars represent the standard error of the mean (s.e.m) for at least two independent experiments.

The *mir-35-41* and *lin-35/Rb* mutant strains both show strongly enhanced RNAi and up-regulation of endo–siRNA targets. Also both strains resemble WT for NRDE-3 localization, which is dependent on siRNAs for nuclear residence (Table 3, Table S1, Figure 2) [21], . These similarities led us to investigate if the *mir-35-41* and *lin-35/Rb* genes might regulate each other. Although *lin-35/Rb* mRNA levels were not significantly different, protein levels of this gene were reduced to about 20% in *mir-35-41(gk262)* relative to wild type worm embryos (Figure 4A and 4B), suggesting that *mir-35-41* positively regulates the accumulation of LIN-35 protein. In contrast, *mir-35* miRNA levels were unaltered in *lin-35(n745)* mutant embryos (Figure 4C). The decreased levels of LIN-35 protein could be responsible for the RNAi hypersensitivity of *mir-35-41* mutants. To test this possibility, we crossed an extrachromosomal array expressing *lin-35* into *mir-35-41(gk262)*. Worms carrying the array, Ex[*lin-35(+); sur-5::GFP*], are distinguished by GFP expression [48]. In the *mir-35-41(gk262)* worms with extra copies of *lin-35* provided by the array (GFP+), the hypersensitivity to *unc-22*(RNAi) was rescued with the majority of worms showing the twitching instead of paralysis phenotype (Figure 4D). The non-GFP (GFP−) siblings were comparable to the original *mir-35-41(gk262)* strain for sensitivity to *unc-22*(RNAi) (Figure 4D). Other GFP transgenes that lack *lin-35* did not affect the RNAi hypersensitivity of *mir-35* mutants, consistent with the idea that *lin-35* is required for the rescue (Figure 4D). The extra copies of *lin-35* also reduced the RNAi hypersensitivity of *lin-35(n745)* genetic mutants (Figure 4D). Rescue of the RNAi phenotype by extra copies of *lin-35* was stronger in *mir-35-41(gk262)* than in *lin-35(n745)*, possibly due to the reduction versus complete absence of LIN-35 protein in these mutants, respectively. To test if extra copies of *lin-35* specifically rescues the RNAi hypersensitivity of *mir-35-41(gk262)*, we introduced the transgene into other enhanced RNAi mutants. The response to *unc-22*(RNAi) was unaffected by the addition of the *lin-35(+)* transgene to *eri-1* or *rrf-3* mutants, supporting the conclusion that the RNAi hypersensitivity of the *mir-35-41* mutants can be attributed to reduced *lin-35/Rb*. A further prediction of this model is that a *mir-35-41;lin-35* double mutant would exhibit the same RNAi response as the *lin-35* single mutant. However, the double mutant proved to be inviable and could not be tested for RNAi sensitivity.

The *lin-35/Rb* gene is a member of the synthetic multivulva B (synMuv B) family, which includes transcriptional repressor and chromatin modifying genes [45]. Worms with combined mutations in synMuv B and synMuv A genes produce multiple vulva structures because of cell lineage defects [49]. In addition to *lin-35*, some of the other synMuv B genes, such as *lin-53*, *lin-9* and *dpl-1*, also negatively regulate the exo–RNAi pathway and cause enhanced RNAi when mutated [22], [23]. We tested if the decreased levels of *lin-35/Rb* in *mir-35-41(gk262)* are sufficient to produce the multiple vulva (Muv) phenotype when combined with the synMuv A mutants *lin-15a(n767)* or *lin-8(n111)*. Double mutants consisting of *mir-35-41(gk262)* and either of the synMuv A genes did not display the Muv phenotype (n = 230 adult worms/strain). In comparison, 100% of the *lin-35(n745)*; *lin-15a(n767)* and the *lin-35(n745)*; *lin-8(n111)* double mutants were Muv (n = 240 adult worms/strain). Taken together, these results suggest that RNAi efficiency is more sensitive than the vulva formation pathway to reduction in *lin-35/Rb* levels or that tissues dependent on *lin-35/Rb* for vulva formation produce sufficient protein in the absence of the miR-35-41 miRNAs.

### Maternal rescue of RNAi hypersensitivity in *mir-35-41* and *lin-35/Rb* mutants

**Figure 5:**
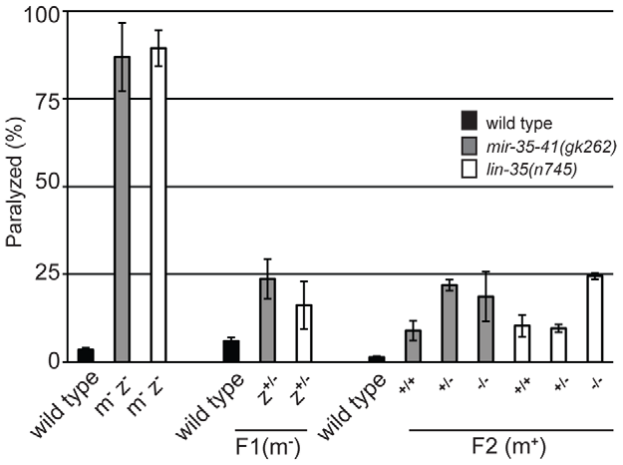
Maternal rescue of RNAi hypersensitivity in *mir-35-41* and *lin-35/Rb* mutants. Histogram representing the percent of paralyzed worms for the indicated strains fed *unc-22* dsRNA for 28 hours from the L4 to adult stage. Wild type and the mutants *mir-35-41(gk262)* and *lin-35(n745)* (maternal^−^, zygotic^−^: m^−^/z^−^) were crossed to wild type males containing a GFP expressing transgene to generate green heterozygous F1 progeny (m^−^/z^+^), which were transferred to *unc-22* RNAi and scored for paralysis. To test for maternal rescue of RNAi sensitivity, heterozygous F1s were singled and allowed to self fertilize. The F2 progeny were transferred to *unc-22* RNAi and each worm was scored for paralysis and then genotyped. Error bars represent the standard error of the mean (s.e.m) for two independent experiments.

Previous work has shown that the miR-35-41 cluster of miRNAs is predominantly expressed in embryos with comparatively little expression in larval and adult somatic tissues [34]. In fact, miRNAs from this cluster make up about 75% of all mature miRNAs present in early embryos [50]. Since the miR-35-41 miRNAs are present in one-cell stage embryos before zygotic transcription by RNA Polymerase II has initiated [50], [51], the miRNAs or their precursors are likely supplied by maternal germ cells. Thus, we tested if maternal contribution of *mir-35-41* activity would be sufficient to regulate RNAi sensitivity. Since our results suggest that the RNAi hypersensitivity of *mir-35-41* mutants is through *lin-35/Rb*, we also analyzed maternal rescue of *lin-35(n745)*. Crossing of *mir-35-41(gk262)* or *lin-35(n745)* hermaphrodites to wild type males rescued the *unc-22*(RNAi) paralyzed phenotype to ∼20% in the F1 progeny compared to ∼80% in the parental strains (Figure 5). These heterozygous F1's were allowed to self-fertilize and the resulting F2 progeny were scored for the paralysis phenotype and then genotyped. Regardless of the zygotic genotype, the worms that had come from mothers with one wild type allele for *mir-35-41* or *lin-35* were less sensitive to *unc-22*(RNAi) than the original mutant strains (Figure 5). Thus, maternal contribution of *mir-35-41* or *lin-35/Rb* is sufficient to regulate RNAi sensitivity.

## Discussion

We have shown that the *mir-35-41* miRNA gene inhibits the exogenous RNAi pathway by positively regulating the expression of LIN-35/Rb protein. The effect of the miR-35-41 miRNAs on LIN-35/Rb levels is likely indirect as miRNAs typically repress gene expression and obvious binding sites for the miRNAs are not present in the *lin-35* sequence. Regulation of LIN-35/Rb appears to be through a post-transcriptional mechanism since mRNA levels were unaffected in *mir-35-41* mutants. We, and others, have been unable to identify direct targets of *mir-35-41* that could explain the mis-regulation of LIN-35 levels, RNAi hypersensitivity, or embryonic lethal phenotypes of *mir-35-41* mutant strains [32]. Positive regulation of the endo–siRNA pathway by *mir-35-41* is likely indirect through targets of this miRNA family. Although few predicted targets of miR-35-41 were up-regulated upon loss of these miRNAs, this result is consistent with a recent study showing that embryonic targets of miR-35-41 undergo deadenylation while the rest of the mRNA remains stable in many cases [36]. Thus, microarrays lack the sensitivity to detect genes mis-regulated in *mir-35-41* mutant embryos. The seven miRNAs in the miR-35-41 cluster share a common seed sequence and may regulate many genes that affect the viability and RNAi sensitivity phenotypes of *mir-35-41* mutants, which could prevent individual targets from being discovered through genetic approaches.

The reason for enhanced exo–RNAi in *lin-35* mutant strains is yet to be fully understood. Consistent with its role as a transcriptional repressor, loss of *lin-35/Rb* activity results in mis-expression of germline specific genes in somatic cells [23], [24], [52]. However, this effect alone cannot explain the enhanced RNAi, as *lin-35* mutants exhibit hypersensitivity in both germline and somatic cells [22], [52]. Several genes for Argonaute proteins that function in the RNAi pathway are also up-regulated in *lin-35/Rb* mutants [24]. Additionally, targets of endo–siRNAs are overexpressed in the absence of *lin-35/Rb*, indicating that the endo–RNAi pathway is defective in *lin-35/Rb* mutants [24]. Thus, the increased expression of RNAi factors and reduced competition with the endo–RNAi pathway may underlie the improved efficiency of RNAi in *lin-35* mutants. We found that sufficient endo–siRNAs are produced to target NRDE-3 to the nucleus in *lin-35/Rb* and *mir-35-41* mutants, suggesting that these genes regulate the function, but not accumulation, of endo–siRNAs. Furthermore, both the spliced and unspliced forms of the endo–siRNA target E01G4.1 were up-regulated in *mir-35-41* mutants. Since RNAi can silence targets at the transcriptional level in worms, *mir-35-41* may also regulate RNAi effectiveness through this pathway [7], [8], [53].

RNAi initiated from exogenous dsRNA can propagate across generations in worms and other organisms [54], [55], [56], [57]. While the Argonaute RDE-1 and dsRNA binding protein RDE-4 are required to activate the RNAi response, they are dispensable for maintenance of RNAi [55], [57]. Instead, chromatin remodeling factors seem to mediate the inheritance of RNAi-induced phenotypes [53], [57]. Thus, the original RNA signal that directs post-transcriptional silencing of complementary targets may also stimulate chromatin remodeling events that repress gene expression across generations. Our results indicate that *mir-35-41*, through regulation of *lin-35/Rb*, decreases exo–RNAi potency and that this effect can be maternally contributed. The RNAi hypersensitivity of *lin-35/Rb* mutants can also be maternally rescued when the gene is expressed from the chromosomal locus but not from a transgene (Figure 4D and Figure 5). The inability of *Ex[lin-35(+); sur-5::GFP]* transgenes to provide maternal rescue has previously been observed and is likely due to the generally poor expression of transgenes in the germline [48], [58].

The *mir-35-41* miRNAs are expressed in oocytes and present in early embryos prior to the onset of zygotic transcription [32], [50]. This expression pattern is consistent with our demonstration that maternal contribution is sufficient to regulate exo–RNAi sensitivity. However, this activity can also be provided by the wave of zygotic expression of *mir-35-41* during embryogenesis [50], since mating of *mir-35-41(gk262)* to wild type worms rescues the RNAi hypersensitivity phenotype. Although the sequences of the *mir-35-41* miRNAs are not conserved across species, many animals express high levels of poorly conserved miRNAs from clusters in embryonic stem cells and during early embryogenesis [38], [59], [60], [61]. One role for these miRNAs is to target maternal mRNAs for degradation [36], [38], [59], [62]. Our results show that miRNAs can also affect the activity of other small RNA pathways, demonstrating a broad function in regulating gene expression through both direct and indirect silencing mechanisms.

## Materials and Methods

### Nematode strains

*C. elegans* worm strains were maintained on NGM plates seeded with OP50 bacteria, under standard conditions [63]. Worms were synchronized by hypochlorite treatment of gravid hermaphrodites followed by overnight hatching of embryos at 20°C. Strains used in this study include the following: wild type (WT) Bristol N2 strain, NL2098 *rrf-1(pk1417)*I, MT10430 *lin-35(n745)*I, VC514 *mir-35-41(gk262)*II, NL2099 *rrf-3(pk1426)*II, MT111 *lin-8(n111)*II, WM49 *rde-4(ne301)*III, GR1373 *eri-1(mg366)*IV, WM158 *ergo-1(tm1860)*V, WM27 *rde-1(ne219)*V, MT1806 *lin-15A(n767)*X, YY158 *nrde-3(gg66)*X. Double mutants: PQ300 [*mir-35-41(gk262);rde-1(ne219)*], PQ301 [*mir-35-41(gk262);rde-4(ne301)*], PQ302 [*mir-35-41(gk262); eri-1(mg366)*], PQ303 [*mir-35-41(gk262);rrf-1(pk1417)*], PQ304 [*mir-35-41(gk262); ergo-1(tm1860)*], PQ421 [*mir-35(gk262);lin-8(n111)*], PQ422 [*mir-35-41(gk262);lin-15A(n767)*], PQ423 [*lin-35(n745);lin-8(n111)*], PQ424 [*lin-35(n745); lin-15A(n767)*], PQ459 [*mir-35-41(gk262);nrde-3(gg66)*]. Non-integrated transgenic strains: PQ20 [*mir-35-41(gk262); apEX160 [mir-35-41]]*, MH1461 [*lin-35(n745); kuEx119 [lin-35(+); sur-5::GFP*]], PQ416 [*mir-35-41(gk262); kuEx119 [lin-35(+), sur-5::GFP*]], PQ439 [*mir-35-41(gk262); Ex [myo-2::GFP)*], PQ456 [*eri-1(mg366); kuEx119 [lin-35(+), sur-5::GFP*]], PQ458 [*rrf-3 (pk1426); kuEx119 [lin-35(+); sur-5::GFP*]]. Integrated transgenic strains: YY174 [*ggIS1* [*nrde-3p::3xflag::gfp::nrde-3*]], YY178 eri-1(*mg366*); [*ggIS1* [*nrde-3p::3xflag::gfp::nrde-3*]], PQ427 [*mir-35-41 (gk262); yy1774* [*ggIS1* [*nrde-3p::3xflag::gfp::nrde-3*]], PQ428 [*mir-35-41(gk262);nrIs20 [sur-5::nls-gfp*]], PD4251 *ccIs4251I [myo-3::Ngfp-lacZ]*.

Worm viability was assayed by synchronizing worms and plating L1 hatchlings at 20°C. Worms were transferred to individual plates at the L4 stage and allowed to lay embryos for 24 h at 20°C or 25°C. Parents were then removed from the plates and embryos were counted. Viable worms were counted 40 hours later. Percent viable progeny represents the number of viable worms/total number of embryos laid.

### RNAi experiments

RNAi plates were prepared using 25 ug/mL carbenicillin and 6 mM IPTG (Isopropyl β-D-1-thiogalactopyranoside, Apex). RNAi clones were obtained from the Ahringer feeding RNAi library [64]. For most experiments synchronized L1 worms were cultured on OP50 until the L4 stage (40 hours) and then transferred to RNAi food. After 28 hours adults were scored for *unc-22*, *lin-1* and *sqt-1* phenotypes. For *pos-1* and *sex-1* RNAi, L1 worms were directly plated on RNAi food and allowed to grow to adults. Parents were then transferred to individual RNAi plates and allowed to lay about 50 embryos. Percent embryonic lethality was calculated as the number of embryos that did not hatch by 40 hours later. For paternal/zygotic rescue experiments, wild-type male worms containing a body muscle GFP marker (PD4251) were crossed to *mir-35(gk262)* (mir-35−/−) mutant hermaphrodites. Parents were removed from the mating plate after 24 hours. Young GFP positive worms represented heterozygous mir-35−/+ F1 cross progeny. F1s were allowed to grow to the L4 stage and 50 GFP positive worms were transferred to *unc-22*(RNAi). F1s were scored for phenotypes 28 hours later. For maternal rescue, GFP positive F1's were transferred to individual plates and allowed to lay self-fertilized F2 embryos. Fifty F2 worms at the L4 stage were transferred to *unc-22*(RNAi). Each worm was scored for phenotypes 28 hours later, lysed and genotyped.

### RNA and protein analyses

Trizol reagent (Invitrogen) was used to extract total RNA from frozen embryo pellets of the wild type (N2) and *mir-35(gk262)* strains. RNA samples were reverse-transcribed and hybridized to arrays following the Affymetrix manufacturer's protocol. Samples from three independent replicates of each strain were hybridized to the Affymetrix GeneChip *C. elegans* Genome Arrays representing 22,500 transcripts. Microarray samples were processed by the UCSD GeneChip Microarray Core. The raw data was normalized and t-statics were computed using R and Bioconductor (www.bioconductor.org) with the “affy” package and Benjamini-Hochberg (BH) correction method for multiple comparisons [65]. RNA levels that changed at least 1.5-fold with a probability of p<0.005 after BH correction were considered significantly different in *mir-35(gk262)* mutants relative to wild-type. Quantitative real-time PCR of reverse transcribed RNA (RT-qPCR) was performed with DNase treated RNA and the primers listed in Table S2. Northern blots to detect mRNAs and miRNAs and Western blots to detect proteins were performed as previously described [66]. The antibody for LIN-35 was provided by the Horvitz lab [45].

## Supporting Information

Figure S1 Temperature sensitive embryonic lethality of *mir-35-41(gk262)* mutants. Percent viable progeny of N2 (wild type) and *mir-35-41(gk262)* at 20°C and 25°C, representing the average number of embryos laid that reached the L4 stage, per parent. Error bars for graphs represent the standard deviation for three independent experiments (n>200 embryos for each condition).
(TIF)

Table S1 Microarray data. (a) Up-regulated genes in *mir-35-41* mutant embryos. Affymetrix probes and corresponding expression changes in *mir-35-41(gk262)* compared to wild type embryos. Gene IDs in column “I” represent the overlap with endo–siRNA targets listed on Sheet 2. Zeros represent non-overlap. (b) Down-regulated genes in *mir-35-41* mutant embryos. Affymetrix probes and corresponding expression changes in *mir-35-41(gk262)* compared to wild type embryos. Gene IDs in column “I” represent the overlap with endo–siRNA targets listed on Sheet 2. Zeros represent non-overlap. (c) Compiled endo–siRNA targets from Lee et al. [18], Ruby et al. [11], Pak et al. [13], Gu et al. [47].
(XLS)

Table S2 List of Primers. Primers used for generation of the *mir-35-41* rescue fragment, for genotyping and for RNA expression analyses.
(DOCX)
