# Supplementary figures and images for: The miR-35-41 Family of MicroRNAs Regulates RNAi Sensitivity in Caenorhabditis elegans
Source: PLoS Genet. 2012 Mar 8;8(3):e1002536. doi: 10.1371/journal.pgen.1002536 (PMC3297572; doi:10.1371/journal.pgen.1002536)

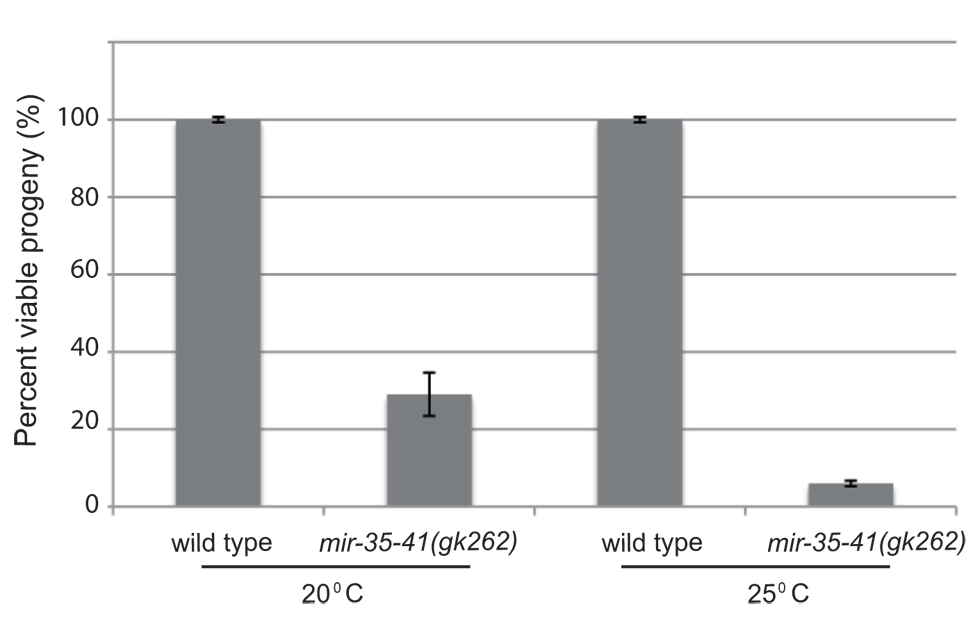

Supplement: Figure S1 — Temperature sensitive embryonic lethality of mir-35-41(gk262) mutants. Percent viable progeny of N2 (wild type) and mir-35-41(gk262) at 20°C and 25°C, representing the average number of embryos laid that reached the L4 stage, per parent. Error bars for graphs represent the standard deviation for three independent experiments (n>200 embryos for each condition). (TIF) [file pgen.1002536.s001.tif]
