# Supplementary material for: The miR-35-41 Family of MicroRNAs Regulates RNAi Sensitivity in Caenorhabditis elegans
Source: PLoS Genet. 2012 Mar 8;8(3):e1002536. doi: 10.1371/journal.pgen.1002536 (PMC3297572; doi:10.1371/journal.pgen.1002536)
Supplement: Table S2 — List of Primers. Primers used for generation of the mir-35-41 rescue fragment, for genotyping and for RNA expression analyses. (DOCX) [file pgen.1002536.s003.docx]

**Supplementary Table 2.** Primers used for genotyping, RNA expression analyses and for generation of the *mir-35-41* rescue fragment.

| Target | Lab designation | Sequence |
| --- | --- | --- |
| *mir-35-41(gk262)* | A68  A69  A122 | GCCACTGCTAGTTTCCACCCGGTGA  GGATCAGATCGAGCCATTGCTGG  TAGGTGATTTTTCACCCGGTGATAGCGAG |
| *lin-35(n745)*  Digest with MnlI | A2076  A2077 | cagacaccgccaccatcacaatc  cgttgaaccgcgtggttgtacgttcc |
| *apEX176* | A418  A762 | CCGAGAACACGATGAACCTTG  CTCGCAGATACTGCTATAGATGAGG |
| mir-35 | A68 | GCCACTGCTAGTTTCCACCCGGTGA |
| E01G4.5 F | A2600 | ACCACGTGGCCAAGCTTTTTG |
| E01G4.5 R intron | A2601 | GTTAAGCTGAAAAATTCGTTTTCTTCA |
| E01G4.5 R exon | A2602 | GTGGTGACCCTCCCCCTCCT |
